# Initial Brazilian experience of Telestroke for thrombolysis in a community hospital

**DOI:** 10.1186/cc12211

**Published:** 2013-03-19

**Authors:** CA Abreu Filho, A Caluza, M Steinman, G Silva, R Cal, N Akamine, J Teixeira, A Andrade, E Silva, A Kanamura, M Cenderoglo, C Lottenberg

**Affiliations:** 1 Hospital Israelita Albert Einstein, São Paulo, Brazil; 2 Hospital Municipal Dr. Moysés Deutsch, São Paulo, Brazil

## Introduction

Stroke is the leading cause of death in Brazil, the number of stroke patients receiving thrombolysis therapy in the developing world is extremely low. Prehospital delay, financial constraints, and lack of infrastructure are the main barriers for thrombolysis therapy in developing countries. In this way, telemedicine (TM) allows knowledge transfer of medical expertise to remote community hospitals, where there is a shortage of specialized doctors. The aim of this study is to describe the first Brazilian initiative of a Telestroke Program for therapeutic support and monitoring cases of acute stroke, and venous thrombolysis of eligible cases in a community hospital in São Paulo, Brazil.

## Methods

Since May 2012 a TM Program has been implemented at two hospitals in São Paulo - Hospital Municipal Dr. Moysés Deutsch (HMMD), a public, secondary hospital, distant about 60 minutes from the nearest tertiary center, and Hospital Israelita Albert Einstein (HIAE), a tertiary private philanthropic entity - due to a partnership with Brazilian Health Ministry. Patients admitted to the community hospital's emergency department with acute ischemic stroke as possible diagnosis are submitted to brain imaging examinations, and considered for remote consultation. A TM Central Command was located at HIAE with Endpoint 97 MXP Cisco^® ^Solution and a mobile Intern MXP ISDN/IP Cisco^® ^for the remote hospital (HMMD). Imaging examinations were evaluated using PACS technology. At HIAE the TM Center has skilled doctors, including neurologists, available for consultation 24 hours, 7 days a week. They discussed the neurological cases via TM, and selected stroke patients for intravenous thrombolysis when timely (3 hours symptom onset).

## Results

HMMD receives an average of 30 cases of stroke monthly, and thrombolysis did not occur before the implementation of the TM Project, because of the lack of neurologists available to conduce the cases. After implementation of the TM Program, six cases of ischemic stroke were thrombolyzed with alteplase; only one case (16%) progressed to death from septic shock, and one case (16%) presented symptomatic intracranial hemorrhage.

## Conclusion

Thrombolysis in ischemic stroke reduces 30% the risk of disability and 18% the mortality rate. This procedure has been only feasible to be done in the community setting because of the implementation of the TM Project, which permits the presence of a real time consultation with a specialized neurological team from a tertiary center.

